# Brain MRI morphometric analysis in Parkinson’s disease patients with sleep disturbances

**DOI:** 10.1186/s12883-018-1092-6

**Published:** 2018-06-20

**Authors:** Andrius Radziunas, Vytenis Pranas Deltuva, Arimantas Tamasauskas, Rymante Gleizniene, Aiste Pranckeviciene, Kestutis Petrikonis, Adomas Bunevicius

**Affiliations:** 1 Neuroscience Institute, Lithuanian University of Health Sciences, Kaunas, Lithuania; 2 Department of Neurosurgery at Kauno klinikos, Lithuanian University of Health Sciences, Kaunas, Lithuania; 3 Department of Radiology at Kauno klinikos, Lithuanian University of Health Sciences, Kaunas, Lithuania; 4 Department of Neurology, Medical Academy, Lithuanian University of Health Sciences, Kaunas, Lithuania

**Keywords:** Parkinson’s disease, Sleep disturbances, Distressing dreams, Nocturnal hallucinations, Nocturia, MRI morphometry

## Abstract

**Background:**

Sleep disturbances are common in patients with advanced Parkinson disease (PD). The aim of this study was to evaluate a possible association of cortical thickness, cortical and subcortical volume with sleep disturbances in PD patients.

**Methods:**

Twenty-eight PD patients (14 men and 14 women, median age 58 years) were evaluated for sleep disturbances with PDSS and underwent brain MRI. Control group consisted of 28 healthy volunteers who were matched by age and gender. Automated voxel based image analysis was performed with the FreeSurfer software.

**Results:**

PD patients when compared to controls had larger ventricles, smaller volumes of hippocampus and superior cerebellar peduncle, smaller grey matter thickness in the left fusiform, parahipocampal and precentral gyruses, and right caudal anterior cingulate, parahipocampal and precentral hemisphere gyruses, as well as smaller volume of left rostral middle frontal and frontal pole areas, and right entorhinal and transverse temporal areas. According to the Parkinson’s disease Sleep Scale (PDSS), 15 (53.58%) patients had severely disturbed sleep. The most frequent complaints were difficulties staying asleep during the night and nocturia. The least frequent sleep disturbances were distressing hallucinations and urine incontinence due to off symptoms. Patients who fidgeted during the night had thicker white matter in the left caudal middle frontal area and lesser global left hemisphere cortical surface, especially in the lateral orbitofrontal and lateral occipital area, and right hemisphere medial orbitofrontal area. Patients with frequent distressful dreams had white matter reduction in cingulate area, and cortical surface reduction in left paracentral area, inferior frontal gyrus and right postcentral and superior frontal areas. Nocturnal hallucinations were associated with volume reduction in the basal ganglia, nucleus accumbens and putamen bilaterally. Patients with disturbing nocturia had reduction of cortical surface on the left pre- and postcentral areas, total white matter volume decrease bilaterally as well in the pons.

**Conclusions:**

PD patients with nocturnal hallucinations had prominent basal ganglia volume reduction. Distressful dreams were associated with limbic system and frontal white matter changes, meanwhile nocturia was mostly associated with global white matter reduction and surface reduction of cortical surface on the left hemisphere pre- and postcentral areas.

## Background

Sleep disturbances are common in Parkinson’s disease (PD) patients. The study by Factors et al. comprised of 78 PD patients with mean Hoehn and Yahr stage of 2.8 (mid stage) found that sleep disturbances occur in up to 90% of patients [1]. Common sleep disturbances in PD are insomnia, restlessness, nocturnal psychosis, nocturia and excessive daytime sleepiness [2]. Good sleep quality is crucial for PD patients’ physical and mental wellbeing [3].

Comprehensive neuroanatomical substrate analysis in relation to multiple sleep disturbances of PD patients is missing. Voxel based morphometry studies usually focus on specific sleep disorders or syndromes but not on patient reported factors associated with sleep disturbances. This creates a scarce picture of neuroanatomical changes associated with multiple perceived sleep disturbances in PD patient.

Rapid eye movement sleep behavior disorder (RBD) affects up to 45–60% [4] of PD patients and is characterized by distressful dreams, moving a lot in the bed, shouting, screaming and fighting with someone in their dreams [5]. PD patients with RBD are at greater risk for cognitive decline [6]. Because of high prevalence and co-morbidity with PD, the RBD has received the most attention in PD [4]. Voxel based morphometry studies of RBD in PD patients revealed morphometric changes in the pontomesencephalic tegmentum, medullary reticular formation, hypothalamus, thalamus, putamen, amygdala and anterior cingulate cortex [7]. Neuroanatomical substrate of the RBD relies on pontomedullary pathway dysonchronisation theory that is responsible for muscle atonia during the rapid eyes movement (REM) phase. Vocal or physical activity during the REM phase can also be due to damage in the basal ganglia [8]. These theories lead to presumption that lesions in the dorsal tegmentum area of the pons and ventral part of the medulla may underlie the RBD [8]. Sleep disturbance and symptoms not meeting the RBD diagnostic criteria are common but remain under-studied in PD population.

Restless legs syndrome (RLS) is another frequent and disturbing complaint of PD patients with prevalence rate reaching 22% [9]. RLS was widely investigated in structural and functional neuroimaging studies [10, 11]. Restlessness at night can be explained by different reasons. For example, bad dreams can cause restlessness in the absence of RBD, pain and urge to urinate during the sleep etc. Possible specific brain morphological features of patients with prominent fidgetiness could be useful in differentiating the underlying reasons of RLS.

Nocturia affects up to 62% of PD patients and has significant adverse effect on quality of life [12]. It develops due to disruption of bladder control loop, which involves cerebral cortex, basal ganglia and micturition center in the pons and spinal cord [13]. Studies analyzing brain morphology in patients with nocturia are lacking.

The aim of this exploratory study was to investigate possible associations of cortical and subcortical brain structures with different factors affecting sleep quality in PD patients.

## Methods

### Subjects

We prospectively recruited 30 PD patients from Departments of Neurosurgery and Neurology of the Lithuanian University of Health Science Hospital Kaunas Clinics, Kaunas, Lithuania, in a period from January, 2015 until September, 2016. Inclusion criteria were (i) idiopathic PD with disease duration of more than 5 years; (ii) good response to L-DOPA therapy; (iii) absence of severe cognitive deficit; and (iv) signed informed consent. Exclusion criteria were (i) current dopamine agonist and psychotropic drug use; (ii) active psychiatric disorder(s); (iii) cognitive impairment (defined as Mini Mental State Examination (MMSE) [14] score < 24) and (iv) structural changes on brain MRI (subtle ischemic or lacunar infarction, brain tumors). As a control group we used age and sex matched 28 healthy controls brain MRI. Patients were excluded from the analyses if semiautomated VBM software (Freesurfer) required manual brain mask correction.

### Study design

The study design and consent procedures were approved by the Ethics Committee for Biomedical Research at the Lithuanian University of Health Sciences, Kaunas, Lithuania. All patients gave signed informed consent prior to inclusion in the study.

Eligible PD patients were instructed about their eligibility to participate in this study. After singing written informed consent form patients underwent evaluation for PD severity (Unified Parkinson disease rating scale motor part III or UPDRS - III [15]), global cognitive functioning (mini mental state examination-MSE [14]), depressive/anxiety symptom severity (Hospital Anxiety and Depression scale (HADS) [16]) and sleep quality (Parkinson disease sleep scale (PDSS) [17]).. During the same admission all patients underwent brain MRI.

### Instruments

#### Motor functioning assessment

PD severity was evaluated using the UPDRS scale [15] that consists of four parts assessing mentation, behavior and mood (Part I); activities of daily living (Part II); motor function (Part III); and treatment complications (Part IV). We used the UPDRS Part III section for evaluation of PD severity during “on” and “off” medication states.

#### Depression assessment

Depressive symptom severity was evaluated the HADS scale that is widely used in clinical practice to assess anxiety (HADS-A) and depression (HADS-D) symptom severity [16]. Lithuanian version of this scale is validated for depression and anxiety screening [18]. Each HADS subscale consists of two 7 item subscales, with score in each items ranging from 0 to 3. Greater score on the HADS-A and HADS-D subscales correspond to greater respective symptom severity.

#### Cognitive function

Mini Mental State Examination (MMSE) [14] was used for initial screening of global cognitive functioning of PD patients. It focusses on attention and calculation, registration, recall, language, repetitions and complex commands. Total MMSE score range from 0 to 30, where higher score means better cognitive function.

#### Sleep quality evaluation

The PDSS [17] was used to assess sleep problems. The PDSS includes 15 visual analogue scale questions usually encountered by PD patients. These questions are designed to evaluate overall quality of night’s sleep (question 1), sleep onset and maintenance insomnia (questions 2 and 3), nocturnal restlessness (questions 4 and 5), nocturnal psychosis (questions 6 and 7), nocturia (questions 8 and 9), nocturnal motor symptoms (questions 10–13), sleep refreshment (question 14) and daytime dozing (question 15). Patients indicate on visual analogue scale frequency and severity of sleep quality disturbing problem*.* Scores on all item are summed giving a total score ranging from 0 (most severe symptom) to 150 (free of symptoms). Total PDSS score below 82 or any item scored less than 5 suggest significant sleep disturbances [19, 20]. All PD patients who completed the PDSS were included in the analyses. Individual PDSS item scores were used for correlations analyses with cortical and subcortical voxel based morphometry (VBM) values.

#### MRI acquisition

All scans were obtained using the 1.5 T Siemens Avanto scanner. The imaging protocol included axial T2W, T1W/mpr/p2/iso and sagittal T2W/spcp2/iso sequences of the entire brain and using the following parameters: T2W: TR 4740 ms; TE 104 ms; 2.0 mm thickness; FoV 250 (192 × 256); concatenation 2, flip angle 120; T2W/spcp2/iso: TR 3200 ms; TE 376 ms; 1.0 mm thickness; FoV 260 (256 × 256); concatenation 1; T1W/mpr/p2/iso: TR 1900 ms; TE 3.35 ms; 1.0 mm thickness; FoV 260 (192 × 256); concatenation 1, flip angle 15. No hardware or software upgrades of the MRI scanner were done during the study period.

#### Image processing and analysis

Automated voxel based subcortical segmentation analyses and cortical parcellation were carried out using the FreeSurfer image analysis software (v6.0, Harvard, MA, https://surfer.nmr.mgh.harvard.edu). Image processing was described previously [21] and demonstrated good test–retest reliability across different MRI scanners [22]. Cortical parcellation provides 34 cortical estimations per hemisphere (based on Killiany/Desikan atlas) [23]. Subcortical segmentation provides 46 region volumes. The automated hippocampal subfield extraction tool outputs left and right volumes of the following structures: presubiculum, subiculum, fimbria, hippocampal fissure, and the tail of the hippocampus. Pons, medulla, superior cerebellar peduncle and whole brainstem volumes were brainstem subfields used for calculation. The output of brain parcellation and segmentation were performed using standard ‘recon-all’ script and all settings were left at default. Subjects who did not successfully finished ‘recon-all’ pipeline were reinspected using the FreeSurfer tool for visualization (Freeview). Two PD patients were excluded from the analyses because skull striping or semi-automated cortical surface or segmentation procedures were not successful. Output for all subjects was thoroughly inspected for segmentation and parcellation errors. Quality checking was aided by scripts supplied by the ENIGMA (Enhancing Neuro-Imaging Genetics Through Meta-Analysis; http://enigma.ini.usc.edu). For further statistical analysis of the data actual values of cortical thickness and volume calculated by Freesurfer were employed.

### Statistical analysis

Data are expressed as median [interquartile range (IQR) 25–75 percentile] and mean ± SD. Normality of data distribution was assessed using the Kolmogorov-Smirnov test. For normally distributed variables parametric two-tailed Pearson test was used. For not normally distributed data non-parametric Spearman test was employed. The SPSS 17.0 (SPSS Inc. Released 2008. SPSS Statistics for Windows, Version 17.0. Chicago: SPSS Inc.) software was used for data analysis. All analyses were performed for the right and left hemispheres separately. The threshold was set at *p* < 0.05 (false discovery rate; FDR) to resolve the problem of multiple comparisons [24]. Brain morphological features and PDSS items which had significant correlation in univariate analysis were adjusted in linear regression model by patient age, gender, MMSE score, Levodopa dosage equivalence, UPDRS-III score and total intracranial volume (ICV). One-way ANOVA was used for comparison of brain morphometric characteristics of PD vs. healthy controls. Significant differences were adjusted for age, gender, MMSE and ICV (general linear model).

## Results

Baseline demographics and clinical characteristics of the study patients are presented in Table 1. Study patients were equally distributed by gender and had no cognitive impairment (Table 1).

**Table 1:** Baseline demographics and clinical characteristics of the study patients and controls

Characteristic	Parkinson’s disease patients	Controls	P value^a^
Age (years)	58 [55–63]	55 [49–65]	0.15
Male/Female (number)	14/14	14/14	0.78
Levodopa dosage equivalence mg	600[400–785]	–	
UPDRS – III (score)	18[12–21]	–	
MMSE (score)	28[26–29]	29[27–30]	0.15^b^
PDSS (score) overall	81.3[64.4–109.9]	–	

^a^Pearson test unless otherwise specified

^b^Spearman test

Values expressed in median [IQR] or number

*UPDRS-III* Unified Parkinson’s Disease Rating Scale, Motor Part III

*MMSE* mini-metal state examination

*PDSS* Parkinson disease sleep scale

### Sleep quality evaluation

According to the total PDSS score, 13 (46%) PD patients had no sleep disturbances. Their mean total PDSS scores were greater than 82 points. The remaining 15 (53.58%) patients had problems with sleep. Detailed analysis of the PDSS showed that the most frequent complaints in this patients group were difficulties staying asleep during the night (median score: 32.5 [4.25–56]) and nocturia (median score: 19 [3–44.5]). The least disturbing sleep problems were distressing hallucinations (median score: 93 [86–98]) and urine incontinence due to off symptoms (median score: 88.5 [42.2–98]).

### Depressive and anxiety symptoms

HADS-D and HADS total scores correlated significantly with scores on the PDSS items of overall quality of night’s sleep (*r* = − 0.47, *p* = 0.01; *r* = − 0.42, *p* = 0.03), sleep onset and maintenance insomnia (*r* = − 0.45, *p* = 0.02; r = − 0.4, p = 0.03), sleep refreshment (*r* = − 0.41, *p* = 0.04; *r* = − 0.49, *p* = 0.008) and total PDSS score (*r* = − 0.44, p = 0.02; r = − 0.44, p = 0.02). Score on the HADS-A subscale correlated significantly with scores on the PDSS items of difficulty staying asleep (r = − 0.44, p = 0.02) and daytime dozing (*r* = − 0.39, p = 0.04), and total PDSS score (r = − 0.41, p = 0.03). The PDSS items that correlated with the HADS-D and/or HADS total scores were excluded from further analysis in order to avoid inclusion of sleep disturbance which might be associated with depression symptoms but not with PD.

### Voxel based morphometry

#### PD vs. controls

PD patients had larger ventricles and smaller volumes of the hippocampus and superior cerebellar peduncle when compared to controls. Grey matter thickness was lower in PD patients relative to controls in three left (fusiform, parahipocampal and precentral) and three right (caudal anterior cingulate, parahipocampal and precentral) hemisphere gyruses. Also, PD patients had lower white matter volume in the left rostral middle frontal area and frontal pole, and in right entorhinal and transverse temporal areas relative to controls.

To define the differences between baseline and brain structure change in PD patients a comparison with healthy normal control was made. (Table 2).

**Table 2:** Comparison of brain morphology characteristics for PD patients and controls

Region	Parkinson’s disease	Controls	Univariate^a^	Adjusted^b^
	Thickness			
	mm ± SD	mm ± SD	(df) = F, p	(df) = F, p
Left caudal middle frontal	2.53 ± 0.19	2.61 ± 0.98	(1,55) = 4.3, 0.04	(1,55) = 0.7, 0.38
Left fusiform	**2.68 ± 0.14**	**2.77 ± 0.95**	**(1,55) = 5.7, 0.02**	**(1,55) = 4.5, 0.03**
Left parahipocampal	**2.61 ± 0.37**	**3.00 ± 0.23**	**(1,55) = 16.3, 0.001**	**(1,55) = 14.4, 0.001**
Left posterior cingulate	**2.37 ± 0.16**	**2.48 ± 0.17**	**(1,55) = 5.0, 0.02**	**(1,55) = 6.1, 0.01**
Left precentral	**2.27 ± 0.19**	**2.41 ± 0.18**	**(1,55) = 7.6, 0.008**	**(1,55) = 4.9, 0.03**
Right caudal anterior cingulate	**2.39 ± 0.30**	**2.56 ± 0.24**	**(1,55) = 4.2, 0.04**	**(1,55) = 4.0, 0.05**
Right isthmus cingulate	2.20 ± 0.16	2.30 ± 0.12	(1,55) = 5.1, 0.01	(1,55) = 2.6, 0.11
Right parahipocampal	**2.67 ± 0.29**	**2.91 ± 0.21**	**(1,55) = 2.0, 0.001**	**(1,55) = 8.3, 0.006**
Right precentral	**2.28 ± 0.22**	**2.41 ± 0.10**	**(1,55) = 5.5, 0.02**	**(1,55) = 4.1, 0.05**
Right superior temporal	2.85 ± 0.13	2.92 ± 0.09	(1,55) = 4.6, 0.02	(1,55) = 3.01, 0.07
Right transverse temporal	2.25 ± 0.25	2.39 ± 0.17	(1,55) = 4.3, 0.04	(1,55) = 3.1, 0.08
	Subcortical structures			
	**mm**^**3**^ **± SD**	**mm**^**3**^ **± SD**	**(df) = F, p**	**(df) = F, p**
Left lateral ventricule	**75.47 ± 31.86**	**153.88 ± 145.9**	**(1,55) = 5.0, 0.02**	**(1,55) = 4.01, 0.05**
Right lateral ventricule	**71.53 ± 27.21**	**132.92 ± 115.21**	**(1,55) = 4.39 0.03**	**(1,55) = 4.4, 0.04**
Right hipocampus total	**42.63 ± 28.8**	**40.41 ± 49.18**	**(1,55) = 5.9, 0.03**	**(1,55) = 6.1, 0.01**
Left hipocampus total	**41.54 ± 38.6**	**39.16 ± 44.0**	**(1,55) = 3.8, 0.05**	**(1,55) = 6.5, 0.01**
Left amygdala	**15.89 ± 22.0**	**14.57 ± 20.03**	**(1,55) = 4.9, 0.03**	**(1,55) = 9.8, 0.003**
	Brainstem structures			
	**mm**^**3**^ **± SD**	**mm**^**3**^ **± SD**	**(df) = F, p**	**(df) = F, p**
Superior cereberal peduncle	**25.84 ± 4.66**	**32.08 ± 9.43**	**(1,55) = 7.0, 0.01**	**(1,55) = 5.4, 0.02**
	White matter volume			
	**mm**^**3**^ **± SD**	**mm**^**3**^ **± SD**	**(df) = F, p**	**(df) = F, p**
Left inferior parietal	9.96 ± 0.13	10.03 ± 0.11	(1,55) = 3.7, 0.05	(1,55) = 3.1, 0.08
Left rostral middle frontal	**9.98 ± 0.07**	**10.04 ± 0.15**	**(1,55) = 4.9, 0.03**	**(1,55) = 4.0, 0.04**
Left superior parietal	10.05 ± 0.05	10.01 ± 0.17	(1,55) = 1.93, 0.05	(1,55) = 1.2, 0.2
Left frontal pole	**9.44 ± 0.25**	**9.64 ± 0.19**	**(1,55) = 8.8, 0.004**	**(1,55) = 5.9, 0.01**
Left insula	9.69 ± 0.07	9.70 ± 0.21	(1,55) = 5.7, 0.02	(1,55) = 3.3, 0.07
Right entorhinal	**8.67 ± 0.21**	**8.87 ± 0.18**	**(1,55) = 11.5, 0.001**	**(1,55) = 10.6, 0.002**
Right transverse temporal	**9.73 ± 0.16**	**9.88 ± 0.19**	**(1,55) = 8.4, 0.005**	**(1,55) = 6.1, 0.01**
Right insula	9.42 ± 0.10	9.52 ± 0.21	(1,55) = 3.7, 0.05	(1,55) = 2.8, 0.09
	Surface area			
	**mm**^**2**^ **± SD**	**mm**^**2**^ **± SD**	**(df) = F, p**	**(df) = F, p**
Left frontal pole	**23.20 ± 2.96**	**25.15 ± 3.51**	**(1,55) = 4.69, 0.03**	**(1,55) = 7.6, 0.008**
Right frontal pole	28.01 ± 3.43	30.05 ± 3.10	(1.55) = 4.62, 0.03	(1,55) = 3.47, 0.06

^a^One way ANOWA analysis

^b^General lineal model adjusted with age, gender, MMSE, ICV

*p* values are FDR corrected

Significant p values are in **bold**

#### Cortical thickness

PD patients had less grey matter in three (fusiform, parahipocampal and precentral) left and three right (caudal anterior cingulate, parahipocampal and precentral) hemisphere than healthy controls after adjustment for age, gender, MMSE and ICV (Table 2).

Nocturnal restlessness correlated negatively with grey matter thickness in the left posterior cingulate gyrus (β = − 0.52, *p* = 0.005) in univariate analysis. However, this correlation was not significant after adjustment for age, gender, MMSE score, Levodopa dosage equivalence, UPDRS-III score and ICV. Scores on other PDSS items did not correlate with thickness of other cortical areas considered in the study.

#### White matter volume

White matter reduction in left hemisphere rostral middle frontal and frontal pole with right hemisphere entorhinal and transverse temporal was found in PD patients as a baseline after adjusting with age, gender, MMSE and ICV (Table 2).

Greater fidgeting during the night was associated with lesser white matter volume of the left hemisphere caudal middle frontal area (β = − 0.64, *p* < 0.0001) in univariate analyses and after adjusting for age, gender, MMSE score, Levodopa dosage equivalence, UPDRS-III and ICV (β = − 0.61, *p* = 0.005). Patients who experienced distressing dreams had white matter reduction in cingulate areas bilaterally (left caudal anterior cingulate [β = 0.54, *p* = 0.003]; left rostral anterior cingulate [β = 0.69, *p* = 0.0001]; right posterior cingulate [β = 0.48, *p* = 0.01] and right rostral anterior cingulate [β = 0.49, *p* = 0.007]) in univariate analyses; however, after adjustment the association remained statistically significant only with the right caudal anterior cingulate (β = 0.45, *p* = 0.04).

Nocturia had strong correlations with left and right hemisphere global white matter volume reduction in univariate analysis and after adjustment (β = 0.29 *p* = 0.01; β = 0.28, p = 0.01) respectively.

#### Cortical surface area

PD patients had surface reduction just in left frontal pole in contrast to healthy controls (Table 2).

**Fig. 1:**
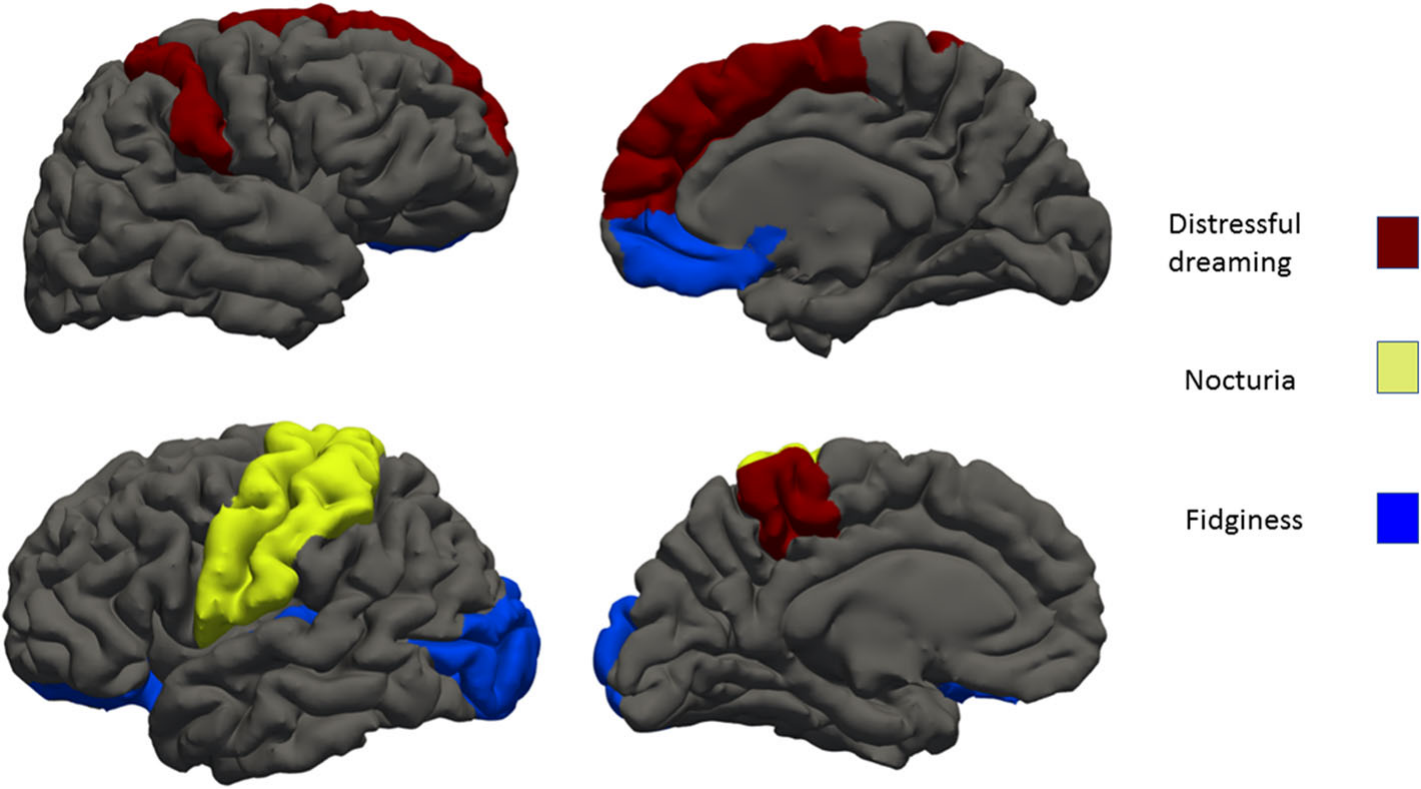
Cortical surface reduction areas with prominent distressful dreaming, fidginess, nocturia

Nocturnal restlessness and distressful dreams had strong correlations with cortical surface reduction (Table 3). Patients who were more fidgeting in the bed during the night had overall surface reduction of the left hemisphere in univariate analysis and after adjustment (β = 0.26, p = 0.04,). Specifically, left transverse temporal gyrus surface (β = 0.47, *p* = 0.01), left lateral orbitofrontal (β = 0.28, p = 0.01) and left lateral occipital (β = 0.49, *p* = 0.02) were significant and after adjustment. These patients also had strong correlation with cortical surface area and on the right hemisphere in medial orbitofrontal area (β = 0.49, *p* = 0.009) on both analysis. Meanwhile, correlations with the right lateral occipital area and superior frontal area lost statistical significance after adjustments (Fig. 1).

**Table 3:** The association of cortical surface areas with the PDDS items 5 (Fidget in the bed) and 6 (Distressing dreams)

	Fidget in the bed		Distressing dreams	
	Univariate β(p)	Adjusted β(p)a	Univariate β(p)	Adjusted β(p)^a^
Left lateral occipital	**0.58 (0.001)**	**0.49(0.02)**	0.12(0.08)	–
Left lateral orbitofrontal	**0.59(0.001)**	**0.28(0.01)**	0.58(0.001)	0.27(0.12)
Left paracentral	0.24(0.07)	–	**0.54(0.003)**	**0.43(0.05)**
Pars obitalis	0.58(0.001)	0.32(0.08)	**0.63(0.0001)**	**0.42(0.01)**
Left tranverse temporal	**0.64 (0.0001)**	**0.47(0.01)**	0.18(0.2)	–
Right lateral occipital	0.53(0.003)	0.36(0.07)	0.09(0.4)	–
Right medial orbitofrontal	**0.59(0.001)**	**0.49(0.009)**	0.21(0.06)	–
Right postcentral	0.24(0.1)	–	**0.56, 0.002**	**0.43(0.01)**
Right superior frontal	0.56(0.002)	0.22(0.11)	**0.55, 0.002**	**0.29(0.03)**
Left hemisphere area total surface	**0.54(0.003)**	**0.26(0.04)**	0.21(0.07)	–

^a^age, gender, MMSE score, Levodopa dosage equivalence, UPDRS-III and intracranial volume

*p* values are FDR corrected

Significant values in both analyses are bold

Distressful dreams had strong correlations with cortical surface reduction in the left frontal lobe: paracentral gyrus (β = 0.43, *p* = 0.05) and pars orbitalis (β = 0.42, *p* = 0.01). These patients also had cortical surface changes on the right hemisphere superior frontal gyrus (β = 0.29, *p* = 0.03) and postcentral area (β = 0.43, p = 0.01).

Nocturia was associated with surface reduction of the left hemisphere postcentral (β = 0.47, *p* = 0.005) and precentral (β = 0.47, p = 0.005) gyruses. These correlations were significant and after adjustment for age, gender, MMSE score, Levodopa dosage equivalence, UPDRS-III and ICV ((β = 0.34, p = 0.01; β = 0.38, p = 0.01) respectively.

#### Subcortical structures

Both ventricules enlargement and both total hippocampus volumes reduction were strongly associated with PD diagnosis comparing it with healthy controls (Table 2).

Distressing dreams were associated with reduced putamen volume on the left and right hemispheres, but lost statistical significance after adjustment. In adjusted analyses, nocturnal hallucinations were associated with lower left and right nucleus accumbens volumes as well as with lower left and right putamen volumes. Nocturnal hallucinations were associated with lower volumes of left and right thalamus and left and right pallidum in univariate analyses, but lost statistical significance in adjusted models (Table 4).

**Table 4:** The association of volumes of subcortical structures with nocturnal hallucinations

Subcortical structures		Nocturnal hallucinations	
		Univariate β(p)	Adjusted β(p)^a^
Right	Accumbens	**0.66(< 0.001)**	**0.42(0.01)**
	Thalamus	**0.58(< 0.001)**	0.16(0.18)
	Putamen	**0.65(< 0.001)**	**0.38(0.01)**
	Pallidum	**0.56(0.002)**	0.26(0.07)
Left	Accumbens	**0.61(0,001)**	**0.45(0.01)**
	Thalamus	**0.64(< 0.001)**	0.24(0.06)
	Putamen	**0.69(< 0.001)**	**0.39(0.01)**
	Pallidum	**0.52(0.004)**	0.26(0.06)

^a^adjusted by age, gender, MMSE score, Levodopa dosage equivalence, UPDRS-III and ICV

*p* values are FDR corrected

Significant values in both analyses are bold

#### Brainstem

Distressing dreams had strong correlation with superior cerebellar peduncle volume in univariate analyses (β = 0.57, *p* = 0.002) and after adjustment for patient gender, age, MMSE score, Levodopa dosage equivalence, UPDRS-III score and ICV (β = 0.38, *p* = 0.02). Distressing dreams and nocturia correlated with pons volume in univariate analyses (β = 0.43, p = 0.02 and β = 0.39, *p* = 0.04, respectively) but lost statistical significant after adjustment. Superior cerebellar peduncle volume reduction was detected and in PD patients comparing with healthy controls (Table 2).

#### Hippocampus segmentation

Hippocampal analysis showed strong association of distressful dreams severity with left (β = 0.56, p = 0.002) and right (β = 0.68, *p* < 0.001) fimbria volumes in univariate analyses that were not significant after adjustment.

## Discussion

More than half of PD patients in our study reported sleep disturbances. Due to multifactorial nature of sleep disruption in PD and in order to find possible brain morphology changes specific to PD patients we analyzed individual sleep symptoms. There were no associations of total PDSS score with analyzed brain regions. However, there was strong association of four specific sleep problems with selected brain regions. Namely, patients who fidget more in the bed during the night had thinner white matter in the left caudal middle frontal area, and reduced cortical surface area of the left lateral orbitofrontal and lateral occipital areas and right medial orbitofrontal area. Patients with frequent distressful dreams had white matter reduction in both sides cingulate area and reduction of superior cerebellar peduncle volume, also had cortical surface reduction in left paracentral area and pars orbitalis of inferior frontal gyrus and on right hemisphere postcentral and superior frontal area. Meanwhile, nocturnal hallucinations were associated with volume reduction of the basal ganglia, specifically in nucleus accumbens and putamen bilaterally.

Nocturia is the most bothersome non-motor symptom of PD patients [25, 26]. Frequent nocturia episodes negatively affect sleep quality and sleep maintenance, mainly due to difficulties staying asleep during the night and increased daytime sleepiness. Pathophysiology of nocturia in PD patients is multifactorial. Urination control is complex and dysregulation can be due to neurodegeneration in cerebral cortex [26], basal ganglia [27] and hypothalamus [28]. In our small study, nocturia was the most frequent sleep-disturbing factor that correlated with white matter reduction in both hemispheres and surface reduction of cortical surface on the left hemisphere pre- and postcentral areas. Despite of lost significance after adjustment, the pons volume reduction in these patients must be inspected in bigger patients group, because micturition reflex center is located at the dorsolateral pontine tegmentum [29]. All these neuroanatomical changes might add more information about pathophysiology of nocturia in PD patients.

Sleep quality improvement was documented after unilateral subthalamic nucleus [30] and pedunculopontine nucleus [31] deep brain stimulation (DBS) and encourage further studies defining neural circuits responsible for non-motor PD symptoms. Previously documented improvement of nocturia symptoms after permanent stimulation of the motor part of the STN [30] and our findings showing the association of nocturia severity with cortical surface reduction on left side pre- and postcentral areas suggest that DBS can decrease frequency of nocturia by modulating this brain area. This assumption is supported by the STN-DBS and dopamine agonist PET study [32], which revealed normalization of activity in pre- and postcentral area as well as pons activation following the STN stimulation. Our finding add another detail to the puzzle for further investigation responsible for nocturia in PD patients.

The observed association between nocturnal hallucinations and reduced basal ganglia volume was not surprising because nucleus accumbens and putamen are included in the limbic system and are responsible for psychiatric symptom production in PD [33] and schizophrenia [34]. Despite the lost significance after adjustment for bilateral putamen involvement, it can be suspected that putamen volume reduction can also be responsible for production of bad dreams These findings allow us to assume that if neurodegeneration starts in the nucleus accumbens then bad dreams turn into hallucinations, which are the hallmark of ongoing progression of cognitive decline [6]. Putamen and nucleus accumbens volume reduction in PD patients complaining of bad dreams should be investigated in larger studies as a possible hallmark of forthcoming cognitive decline.

Our findings of cingulate involvement in distressful dream generation support the theory of Levin & Nielsen, 2007 that suggests that the anterior limbic system is central to nightmares, because the anterior cingulate has been implicated in pain distress, social exclusion, and separation anxiety and in processing of negative emotional stimuli. The documented white matter reduction in the right cerebellar hemisphere and superior cerebellar peduncle adds more information to before mentioned theory. Although we didn’t analyzed the character of distressful dreams, but cerebellar involvement suggests that cerebellum could be the generator of distressful dreams.

We found previously well-described differences in brain morphometry associated with PD, including neocortical [35] and limbic lobe artrophy [36] and was associated with not cognitive impaired PD. These findings suggest that our VBM analyses results is associated with brain morphometric changes, which can be related to sleep quality.

Although RBD was not specifically evaluated in patients, but our findings suggest that superior cerebellar peduncle, putamen and nucleus accumbens volume reduction might be an important indicator of the RBD. Volume reduction in the aforementioned areas should be further explored as a possible hallmark of RBD.

### Limitations and strengths of the study

Small sample size and lack of objective evaluation of sleep disturbances are the major limitations of this study. For example, the cause of nocturnal restlessness (RBD vs. RLS) can be difficult to differentiate without polysomnography, or specific questionnaires. But our study aim was to analyze patient reported sleep disturbing factors instead of sleep syndromes. The strengths of the study are strict inclusion criteria in terms of drugs used for sleep disturbances, and mood and cognitive assessments. This is the first study focusing on sleep disturbing factors but not on RBD or RLS in PD patients.

## Conclusions

Sleep disturbances are common in PD. Nocturia is the most disabling symptom in PD that is associated with cortical surface reduction in pre- and postcentral areas and lower white matter volume on both hemispheres and pons volume. Distressful dreams are associated with white matter reduction in both sides cingulum as well as superior cerebellar peduncle and right cerebellar hemisphere. Patients with nocturnal hallucinations had marked nucleus accumbens and putamen volume reduction. Further studies exploring potential association of brain morphometric parameters with sleep disturbances of PD patients are encouraged.

## Abbreviations

DBS: Deep brain stimulation
HADS: Hospital Anxiety and Depression scale
ICV: Total intracranial volume
L-DOPA: Levodopa
PD: Parkinson’s disease
PDSS: Parkinson disease sleep scale
RBD: Rapid eye movement sleep behavior disorder
REM: Rapid eyes movement
RLS: Restless legs syndrome
STN-DBS: subthalamic nucleus deep brain stimulation
UPDRS – III: Unified Parkinson disease rating scale motor part III
VBM: voxel based morphometry
